# Functional connectivity is linked to working memory differences in children with reading learning disability

**DOI:** 10.1186/s12887-024-04791-2

**Published:** 2024-05-08

**Authors:** Rodrigo Flores-Gallegos, Thalía Fernández, Sarael Alcauter, Erick Pasaye, Lucero Albarrán-Cárdenas, Bertha Barrera-Díaz, Paulina Rodríguez-Leis

**Affiliations:** Departamento de Neurobiología Conductual y Cognitiva, Instituto de Neurobiología, Universidad Nacional Autónoma de México, Campus Juriquilla, Blvd. Juriquilla 3001, Juriquilla, Querétaro 76230 México

**Keywords:** Reading learning disability, Reading disorder, Functional connectivity, Resting-state fMRI, Working memory

## Abstract

Reading learning disability (RLD) is characterized by a specific difficulty in learning to read that is not better explained by an intellectual disability, lack of instruction, psychosocial adversity, or a neurological disorder. According to the domain-general hypothesis, a working memory deficit is the primary problem. Working memory in this population has recently been linked to altered resting-state functional connectivity within the default mode network (DMN), salience network (SN), and frontoparietal network (FPN) compared to that in typically developing individuals. The main purpose of the present study was to compare the within-network functional connectivity of the DMN, SN, FPN, and reading network in two groups of children with RLD: a group with lower-than-average working memory (LWM) and a group with average working memory (AWM). All subjects underwent resting-state functional magnetic resonance imaging (fMRI), and data were analyzed from a network perspective using the network brain statistics framework. The results showed that the LWM group had significantly weaker connectivity in a network that involved brain regions in the DMN, SN, and FPN than the AWM group. Although there was no significant difference between groups in reading network in the present study, other studies have shown relationship of the connectivity of the angular gyrus, supramarginal gyrus, and inferior parietal lobe with the phonological process of reading. The results suggest that although there are significant differences in functional connectivity in the associated networks between children with LWM and AWM, the distinctive cognitive profile has no specific effect on the reading network.

## Introduction

### Reading learning disability

Reading learning disability (RLD) is characterized by a significant difficulty in reading that manifests as imprecise or slow and effortful word reading, difficulties with spelling, and difficulties understanding the meaning of what is read compared to people of the same age and education level (scores less than 1.5 standard deviations below the mean in specific reading tests) [1]. In the literature, children with RLD are usually described as having dyslexia; however, not all children with RLD have dyslexia because only some have reading comprehension difficulties. RLD includes difficulties in reading accuracy, reading comprehension, and reading speed; according to the American Psychiatric Association [1], children can present difficulties in one, two, or all domains. RLD is more common in children in the early years of primary school; it is not better explained by intellectual disabilities, neurological disorders, psychosocial adversity, or the type of education program [2]. Children with RLD are more likely to present with anxiety and isolation [3], and it has been related to school desertion, low self-esteem, and childhood depression [4].

### Domain-general vs. domain-specific hypotheses

Despite efforts to elucidate the causes of cognitive deficits in children with learning disorders, there is no consensus framework [5]. There are two main hypotheses about atypical processing in RLD. The first hypothesis, the domain-specific cognitive deficit hypothesis, refers to the presence of learning disorder subgroups with specific deficits. Supporting this hypothesis, Siegel reported that RLD subgroups exhibited distinct characteristics that consistently predicted patterns of RLD. Such deficiencies were morphological, semantic, or syntactic in nature, including lexical access. Under the domain-specific view, Landerl [6] reported that dyslexic children showed specific difficulties in phonological processing, which is a specific constraint limiting access to phonological representations and affecting the sequential recall of words and pseudowords. Brandenburg [7] proposed a 5-profile model in which there is no dominant profile of learning disorders. This is consistent with the idea that the cognitive deficits associated with learning disorders are multifactorial and are not the same for all children.

The second hypothesis, the domain-general hypothesis, refers to a limited capacity of attentional control and an impairment in executive functions, particularly in working memory (WM) [8], specifically in the central executive and the phonological loop in Baddeley’s model of WM [9, 10]. The domain-general hypothesis is supported by the finding that children with learning disabilities not only present deficits specific to their academic performance but also present attentional and WM impairment compared to typically developing (TD) children [11]. Moreover, the mistakes made by children with learning disabilities during graphical codification are not due to a phonological deficit but rather a visual attention deficit within the magnocellular pathway [12], which could explain their sluggish attentional shifting ability, along with other deficits in sensory and cognitive domains [13]. The multiple disorders observed in dyslexic subjects do not have to be mutually exclusive or form a single causal chain; some of them may not even be related to the reading problem but simply be correlated with it.

On the other hand, behavioral evidence supporting the domain-specific hypothesis was obtained in a comparison of children with dyslexia, children with other special educational needs (dyspraxia, attentional deficits, language deficits, and behavioral difficulties), and TD children; both the dyslexia and special education groups had lower scores in phonological tasks than the control group; however, the special education group showed a deficit in WM and visuospatial ability [14].

### Working memory

WM is a cognitive system that allows short-term memory storage and the simultaneous manipulation of this information while an individual is performing a complex cognitive task, such as reasoning or learning [15–18]. The information is maintained in consciousness or in the span of attention until it needs to be used for processing; depending on the stage of the process, it is continually updated [19]. The dynamic nature of WM is contrasted with the passive short-term memory system [20]. Baddeley and Hitch [15] proposed a model in which a supervisory system, called the central executive, regulates two temporary memory slave systems, that are responsible for short-term maintenance of domain-specific (e.g., verbal, numerical, or visuospatial) information. The two slave systems are the phonological loop and the visuospatial sketchpad. Both slave systems interact through an episodic buffer to manipulate episodic long-term memory.

The WM system has been linked with functional connectivity, which is the correlation of low-frequency fluctuations in blood oxygen level-dependent (BOLD) signal across different brain regions; more specifically, fluctuations of activity in the salience network (SN), default mode network (DMN), and frontoparietal network (FPN). Although WM has been considered a function of the SN, it is more likely that executive functions, such as WM, result from the interaction among the SN, DMN, and FPN [21]. The SN includes the anterior cingulate cortex, anterior insula, rostral prefrontal cortex, and supramarginal gyrus [22]. The DMN encompasses the posterior cingulate cortex, middle prefrontal cortex, angular gyrus, and middle temporal cortex. Fang et al. [23] analyzed the resting-state connectivity of 264 young adults and the correlation of such activity with performance on an n-back WM task. The researchers found significant moderate correlations of the connectivity between the dorsolateral prefrontal cortex (dLPFC) and the anterior cingulate cortex and between the dLPFC and the fronto-insular cortex with performance on the active working memory task. The mentioned areas are cortical structures in the SN and FPN, suggesting that there is causal interconnectivity between both resting-state networks and WM performance. A longitudinal study conducted by Horowitz-Kraus et al. [24] revealed that the DMN is related to narrative comprehension during childhood, and there is a general deactivation of the DMN during a narrative comprehension task in individuals between 11 and 18 years of age that could be due to functional brain specialization and reduced need for compensatory mechanisms; narrating comprehension is linked with passive working memory. In addition, the FPN contains the dlPFC, inferior parietal lobe, and inferior parietal sulcus [16]. A study by Ostby, Tamnes, Fjell and Walhovd [25] containing 108 participants ranging between 8 and 19 years reported associations of the radial diffusivity of the superior longitudinal fasciculus (SLF) and cortical thickness of the supramarginal gyrus with WM performance on a digit span task. Furthermore, differences in the impact of SLF diffusivity and supramarginal gyrus thickness were found with participant age. Based on structural equation modeling of data from 158 participants between 7 and 18 years old, there was a direct influence of age on processing speed that positively affected WM, which is highly correlated with fluid and crystalized intelligence [26].

### WM in individuals with RLD

There are numerous reports linking poor reading performance with lower WM scores [19, 27–32]. Some authors believe that WM is more important for advanced reading skills, such as reading comprehension [29], while others posit that WM is more important for developing reading skills, as in younger children [20].

WM can include verbal, numerical, or visuospatial information or combinations of these domains. Several authors have suggested that verbal WM is more closely related to reading than numerical WM [33] or visuospatial WM [34]. However, in a meta-analysis, Peng et al. [20] concluded that there is a strong relationship between reading and WM in younger readers (before 4th grade) and that this relationship does not depend on the specificity of WM.

Resting-state functional magnetic resonance imaging (fMRI) revealed a strong correlation of activity in the SN and the DMN with that in the reading network (RNW), defined by the functional connectivity between different brain regions related to reading performance [35] in young children [36]. A study by Twait et al. [22] compared the functional connectivity of children (31 with developmental dyslexia (DD) and 35 TD children) during a reading comprehension task; children with DD had lower general connectivity of the SN than TD children. Regions of interest (ROIs) were based on the atlas provided in the CONN toolbox. Another resting-state fMRI study showed that children with DD had higher functional connectivity between the right visual association areas and right prefrontal attention areas than TD children [37]. Moreover, there is evidence that reading interventions for children with reading difficulties increase the connectivity of the SN and the cingulo-opercular network (CON) [38]. CON activation was strongly related to FPN activation and top-down control of executive functions [39]; however, the CON was more closely related to tonic alertness, while the FPN was more closely related to executive functions [40].

In an fMRI n-back task, Bailey [35] observed that children with DD who had a lower response rate to a sound-symbol letter correspondence treatment exhibited weaker connectivity among the dlPFC, supramarginal gyrus, inferior frontal gyrus, and mid-frontal cortex than children who had a higher response rate. Furthermore, there is behavioral evidence that children with DD with poor pseudoword reading ability had a longer response time in an extraneous attention task than children with DD with good pseudoword reading ability and individuals in two control groups, one matched by age and the other by reading level [41].

The presented evidence suggests that although children with RLD share the same clinical indicators [1], some of them have greater WM impairments than others. This raises the following question: Are WM differences in children with RLD associated with differences in brain functional connectivity? To our knowledge, no study has addressed this question. The aim of this research was to compare the resting-state functional connectivity of children with RLD and lower-than-average WM with that of children with RLD and average WM. We hypothesized that children with lower WM would have weaker connectivity of WM-related and reading-related regions than matched individuals with average WM.

## Methods

### Ethical consent

A verbal and written explanation of the research and procedure was given to all the participants and their parents. An informed consent form was signed by every participant’s parent or legal guardian and signed by the child. The study guaranteed the confidentiality of the collected data, adhered to the ethical standards of the latest revision of the Declaration of Helsinki (Brazil 2013) and was approved by the Ethics Committee of the Institute of Neurobiology of the Universidad Nacional Autónoma de México (UNAM) [INEU/SA/CB/146].

### Sample

**Fig. 1:**
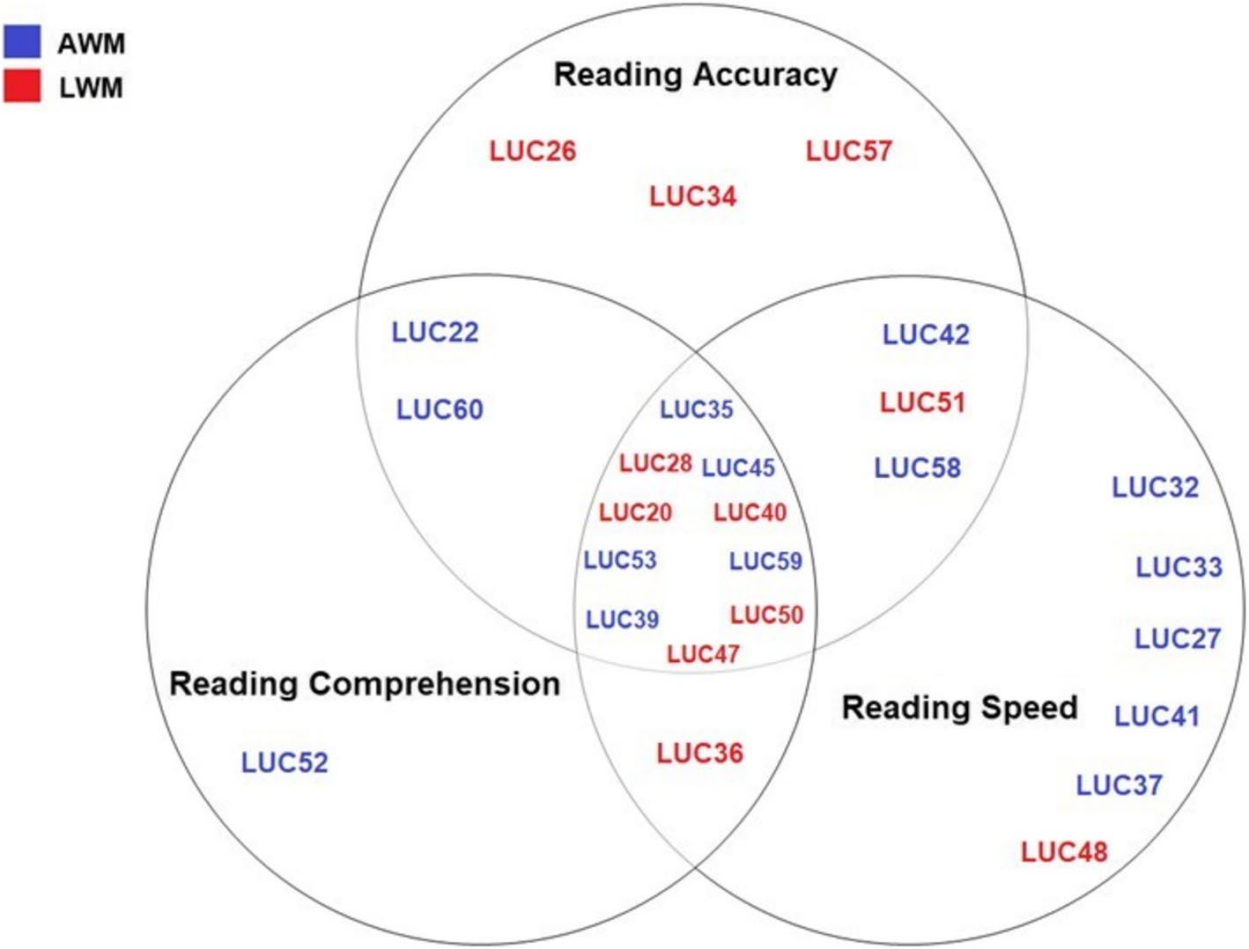
Venn diagram showing the distribution of the areas of difficulty within the reading domain for the lower-than-average working memory group (shown in red) and average working memory group (shown in blue)

Twenty-six right-handed children with RLD who attended first or second grade in public primary schools in Querétaro, México, were included in this study. Based on the DSM-5 [1], all participants had a diagnosis of RLD as they met the following criteria: 1) intellectual coefficient (IQ) equal to or above the standard score of 75; 2) no physical impairments, such as of vision or hearing (need for hearing aids); 3) no other psychiatric disorders, such as ADHD, according to the assessment of a neuropediatrician and psychologist; 4) scores two standard deviations below the mean (i.e., 9th percentile or below) in at least one of the reading areas of the Children Neuropsychological Scale [42] which measures reading accuracy, reading comprehension, and reading speed. The Children Neuropsychological Scale includes several reading tasks, such as syllable, nonword, and word reading; sentence and paragraph reading comprehension; and silent and active reading duration. This scale is standardized for the Mexican population. The participants in this study exhibited heterogeneity in reading domain difficulties; most children (*n* = 10) showed significant difficulties (below 1.5 standard deviations from the mean) in all three domains, 3 children showed difficulty in reading accuracy, 1 child in reading comprehension, 6 children in reading speed, 2 children in reading accuracy and reading comprehension, 3 children in reading accuracy and reading speed, and 1 child in reading comprehension and reading speed, as shown in Fig. 1. According to the Wechsler Intelligence Scale for Children [43], the participants had an IQ of 75 or above (mean = 90.12; range = 75–115). All had normal neurological examination results without a history of brain injury, and they did not present any other psychiatric disorder beyond RLD, according to the results of a neuropediatric evaluation or the Mini-International Neuropsychiatric Interview [44].

The participants were divided into two groups depending on their WM index on the WISC-IV. We use the WM index from the WISC-IV as a proxy for WM. On the one hand, the relationship between WM and reading is stronger in younger children [20], and this relation does not depend on the WM type; on the other hand, the majority of the children in this study, who had RLD exhibited reading processing similar to that of younger children. Taking these considerations into account, it was appropriate to evaluate WM in this way, although the WM index is a composite measure that includes the scores from direct-digits and letters-and-numbers tests, which assess numeric and verbal WM, respectively.

Children with a WM index between 85 and 115 were placed in the average working memory (AWM) group, and children with a WM index between 70 and 84 were placed in the lower-than-average working memory (LWM) group. The AWM group comprised 15 participants (5 females), with a mean age of 8.53 years (range: 7.31–11.72 years), and the LWM group comprised 11 participants (2 females) with a mean age of 9.57 years (range: 7.64–11.53 years). Children in the AWM group align with the domain-specific hypothesis of a specific impairment in reading but not in other domain-general cognitive functions. In contrast, children in the LWM group align with the domain-general hypothesis where an impairment in reading is linked to a deficit in domain-general cognitive functions, such as WM.

According to the Mann–Whitney U test, there were no significant differences between groups in terms of the processing speed index, verbal comprehension index, or perceptual reasoning index of the WISC-IV; however, there was a statistically significant difference between groups in the WM index (AWM average = 94.40; LWM average = 79.72; W = 0; *p* = 0.001), IQ (AWM average = 94.86; LWM average = 83.63; W = 25.50; *p* = 0.003) and age (AWM average = 8.53; LWM average = 9.57; W = 124; *p* = 0.033). The behavioral scores are shown in Table 1. The AWM (females = 33.33%) and LWM groups (females = 18.18%) had different sex distributions (chi-square = 0.74; *p* = 0.389), although the difference was not significant. No differences were found on reading subtests of the ENI-2 (reading accuracy, reading comprehension, and reading speed). In Fig. 1, the red and blue points represent children in the LWM and AWM groups, respectively; children in both groups exhibited a wide variety of deficits in the areas of the reading domain.

**Table 1:** Scores on the Children Neuropsychological Scale (ENI-2) reading area and Wechsler Intelligence Scale for Children (WISC-IV)

Test	Variable	Group	N	mean	SD	SE	W	*P*
**WISC-IV (normalized scores)**	Intellectual Coefficient	LWM	15	83.63	6.59	1.98	25.20	0.003
		AWM	11	94.86	8.95	2.31		
	Verbal Comprehension Index	LWM	15	86.63	7.73	2.33	54.00	0.144
		AWM	11	94.86	13.69	3.53		
	Perceptual Reasoning Index	LWM	15	92.63	11.86	3.57	59.00	0.231
		AWM	11	100.53	15.90	4.10		
	Working Memory Index	LWM	15	79.72	3.13	0.94	0.00	0.001
		AWM	11	94.40	4.64	1.19		
	Processing Speed Index	LWM	15	88.63	8.89	2.68	71.00	0.563
		AWM	11	93.40	12.57	3.24		
**ENI-2 (percentile scores)**	Reading Accuracy	LWM	15	5.72	9.09	2.74	77.50	0.805
		AWM	11	8.71	11.75	3.03		
	Reading Comprehension	LWM	15	19.80	28.67	8.63	69.50	0.513
		AWM	11	17.87	21.32	5.05		
	Reading Speed	LWM	15	12.94	22.14	6.67	89.50	0.726
		AWM	11	6.57	7.89	2.03		

### Procedure

The participants completed a behavioral evaluation within the Laboratory of Psychophysiology at the Institute of Neurobiology of the Universidad Nacional Autónoma de México. The behavioral evaluation consisted of completing the ENI-2 and WISC-IV scales and undergoing a clinical interview and neuropsychiatric evaluation to identify if the participants met the inclusion criteria.

#### Behavioral instruments

##### Children Neuropsychological Scale (ENI-2) [42]

This scale explicitly evaluated reading, writing, arithmetic, and visual attention abilities.

##### Wechsler Intelligence Scale for Children (WISC-IV)

This scale was used to assess the general indices of verbal comprehension, perceptual reasoning, the WM index, processing speed, and IQ [43].

#### fMRI data acquisition

To estimate the functional brain connectivity of the AWM and LWM groups, each participant underwent resting-state fMRI with their eyes closed.

fMRI data were acquired at the National Laboratory of Magnetic Resonance Imaging (*Laboratorio Nacional de Imagenología por Resonancia Magnética;* LANIREM) located at the Instituto de Neurobiología, Universidad Nacional Autónoma de México. A 3 Tesla magnetic resonance imaging (MRI) scanner (GE Healthcare systems, Discovery MR750 3.0 T) was used for this study. fMRI data were acquired with a T2*-weighted echo-planar imaging sequence (repetition time (TR) = 2000 ms, echo time (TE) = 40 ms, and voxel size = 4 × 4 × 4 mm^3^), with 300 volumes obtained in a 10-min acquisition period. A T1 reference image was obtained for anatomical reference using an SPGR sequence with a spoiled gradient of 1 × 1 × 1 mm^3^ (TR = 8.1 ms, TE = 3.2 ms, and spin angle = 12.0°).

In total, including localization, functional and anatomical sequences, this study lasted approximately 30–40 min per participant.

The preprocessing of the data was the same as that used by Gracia-Tabuenca et al. [45]. Preprocessing was performed with FMRIB’s Software Library FSL v.5.0.6 [46]. Briefly, preprocessing included eliminating the first four volumes, temporal spacing, head movement correction, brain extraction, regression of the confounding variables, bandpass filtering (0.01–0.08 Hz), and spatial normalization. The confounding variables were the six parameters from rigid-body motion correction, the average signal from the white matter, cerebrospinal fluid, and the derivatives of the eight mentioned variables, for a total of 16 variables. Finally, each volume was coregistered with the corresponding T1 image and an additional nonlinear registration to the Montreal Neurological Institute (MNI) space.

### Analysis

**Fig. 2:**
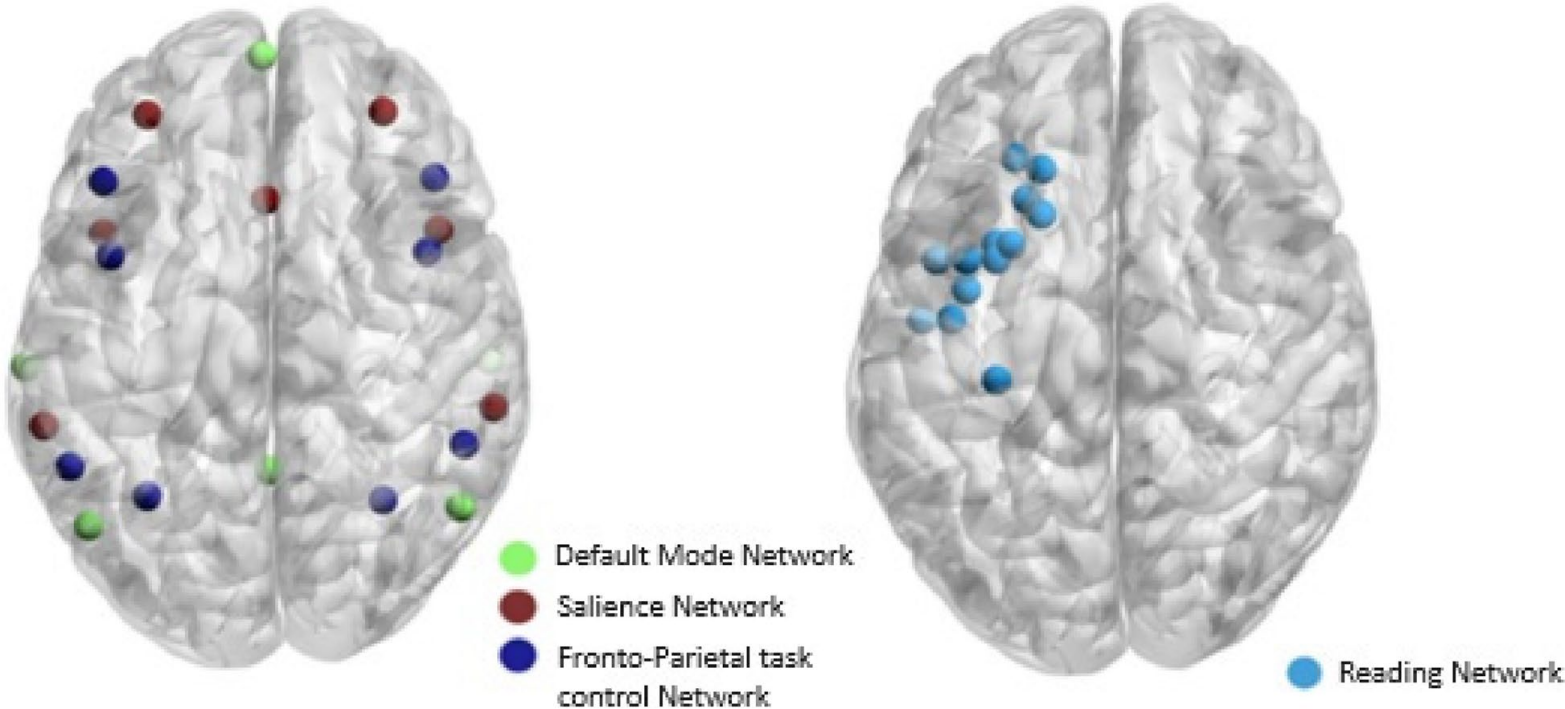
The regions of interest (ROIs, 4 mm spheres) in the DMN (green), SN (red), FPN (dark blue), and RNW (light blue) visualized with BrainNet Viewer [48]

From each resting-state fMRI session, the average signal was extracted from each ROI. The analysis considered the interaction among the SN, DMN, and FPN following Goulden et al. [21] and the evidence in this specific population described in the introduction. Each ROI consisted of a 4 mm sphere that represented a cortical and subcortical structure that belonged to the SN [22], DMN, and FPN [26] (Fig. 2). A separate analysis was performed with the ROIs corresponding to the RNW [36]. The selection of the specified reading network was based on the study by Alcauter et al. [36] which provide resting-state fMRI evidence of a reading network in control children. The ROIs were centered at the coordinates reported in Table 2. Pearson’s correlation coefficients of the relationship between the average signal from all the possible ROI-to-ROI pairs were calculated for each subject, yielding a connectivity matrix, and Fisher’s-z transformation was subsequently applied. The network-based statistics (NBS) approach [47, 48] was applied, using age as a confounding variable, based on the possible impact of age on brain structure and connectivity and WM performance [25, 26] to identify networks (sets of joint connections or clusters of connections) with significant differences between the two groups. In brief, the strength of the clusters of connections that were significantly different (at an initial statistical threshold of t = 2.5) between the two groups was compared with a null distribution of the maximal strength of the components (clusters of connections) that surpassed the same initial statistical threshold using randomly permuted data (here, 10,000 permutations). Based on this null distribution, only clusters with probability of being a false positive lower than 5% were reported. The NBS approach involves multiple mass comparisons, considering the internal structure of the connectivity networks, enabling the construction of general linear models with permutation tests. This procedure naturally explores the network differences between groups of patients and controls adjusting for multiple comparisons using permutation methods. This approach was implemented using the Network-Based Statistics toolbox (NBS v1.2) [47] for MATLAB (version R2019a). Post hoc analyses were performed to characterize the individual connections of each network or set of connections showing significant differences.

**Table 2:** Regions of interest (4 mm-radius spheres) of the explored functional brain networks

Areas	Network	MNI Coordinates Sphere Center		
		**x**	**y**	**z**
Dorsolateral Prefrontal Cortex (r)	Frontoparietal task control Network (Power et al. 2011) [16]	46	28	31
Dorsolateral Prefrontal Cortex (l)		-44	27	33
Frontal Lobe (r)		44	8	34
Frontal Lobe (l)		-42	7	36
Inferior Parietal Lobe (r)		54	-44	43
Inferior Parietal Lobe (l)		-53	-50	39
Inferior Parietal Sulcus (r)		32	-59	41
Inferior Parietal Sulcus (l)		-32	-58	46
Posterior Cingulate Cortex	Default Mode Network (Power et al. 2011) [16]	1	-51	29
Middle Prefrontal Cortex		-1	61	22
Angular Gyrus (l)		-48	-66	34
Angular Gyrus (r)		53	-61	35
Lat_tempus Middle Temporal Gyrus (l)		-65	-23	-9
Lat_tempus Middle Temporal Gyrus (r)		61	-21	-12
Anterior Cingulate Cortex	Salience Network (Twait et al. 2018) [22]	0	22	35
Anterior Insula (l)		-44	13	1
Anterior Insula (r)		47	14	0
Rostral Prefrontal Cortex (l)		-32	45	27
Rostral Prefrontal Cortex (r)		32	46	27
Supramarginal Gyrus (l)		-60	-39	31
Supramarginal Gyrus (r)		62	-34	31
Caudate (l)	Reading Network (Alcauter et al. 2017) [36]	-10	14	8
Insula (l)		-22	22	-8
Lat_Middle Frontal Gyrus		-26	10	36
Middle Temporal Gyrus (l)		-50	-10	-4
Operculum Inferior Frontal Gyrus (l)		-30	6	32
Orbital Inferior Frontal Gyrus (l)		-25	33	-8
Putamen (l)		-18	18	0
Rolandic Operculum (l)		-38	6	20
Dorsolateral Superior Frontal Gyrus (l)		-18	30	36
Superior Temporal Gyrus (l)		-46	6	0
Superior Temporal Pole (l)		-46	6	4
Transverse Temporal Gyrus (l)		-30	-26	12
Area Triangularis (l)		-30	10	28
Postcentral Gyrus (l)		-42	-10	40
Precentral Gyrus (l)		-38	-2	36

*(r)* Right hemisphere, *(l)* Left hemisphere

In a secondary analysis, to explore the possibility of an association among cognitive functions (WM, processing speed, perceptual reasoning, and verbal comprehension), reading ability (accuracy, speed, and comprehension) and the connectivity strength of the identified subnetworks, linear regression analysis was performed with the cognitive and reading variables as dependent variables. The variables group, sex, and network connectivity strength were considered independent variables. The analysis was corrected for multiple comparisons using false discovery rate (FDR).

## Results

### Is there a significant difference in the functional connectivity strength between groups?

**Fig. 3:**
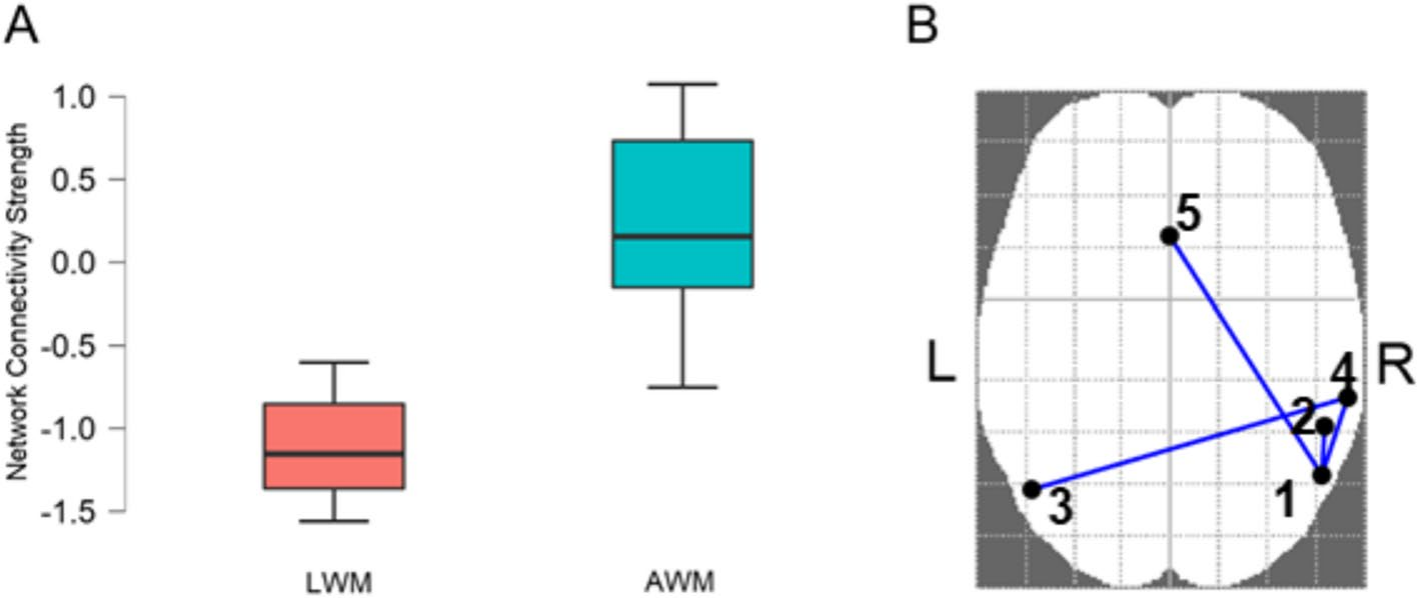
**A** Connectivity strength of the lower-than-average working memory (LWM) group (red) and the average working memory (AWM) group (blue) for the network shown in **B**. **B** Network (cluster of connections) with significant differences in connectivity strength between the LWM and AWM groups, as shown in **A**. The brain regions in this network include the 1) right angular gyrus, 2) right inferior parietal lobe, 3) left angular gyrus, 4) right supramarginal gyrus, and 5) anterior cingulate cortex

NBS revealed a subnetwork with higher functional connectivity strength (*p* = 0.034; Fig. 3) in the AWM group (mean = 0.196; SD = 0.610) than in the LWM group (mean = -1.097; SD = 0.340). This subnetwork was formed by connections among the right angular gyrus (rAG), left angular gyrus (lAG), right supramarginal gyrus (rSMG), right inferior parietal lobe (rIPL), and the anterior cingulate cortex (ACC). For more detail on ROI connectivity, see Table 3. No significant connectivity strength differences in the specified RNW in this paper were found between the groups.

**Table 3:** Bivariate connectivity between regions of interest of the resulting network cluster for the lower-than-average working memory group (LWM) group (red) and average working memory (AWM) group (blue)

Regions of Interest		Group	Connectivity	SD	t	Cohen-d	Power
Angular Gyrus (r)	Supramarginal Gyrus (r)	LWM	-0.365	0.174	3.72	1.47	0.94
		AWM	0.023	0.383			
Angular Gyrus (r)	Anterior Cingulate Cortex	LWM	-0.423	0.204	3.40	1.35	0.90
		AWM	-0.139	0.194			
Inferior Parietal Lobe (r)	Supramarginal Gyrus (r)	LWM	0.194	0.283	2.76	1.09	0.75
		AWM	0.450	0.290			
Angular Gyrus (l)	Supramarginal Gyrus (r)	LWM	-0.503	0.250	2.83	1.12	0.77
		AWM	-0.138	0.300			

*(r)* Right hemisphere, *(l)* Left hemisphere

### Are there associations of network connectivity strength with cognitive and reading variables?

**Fig. 4:**
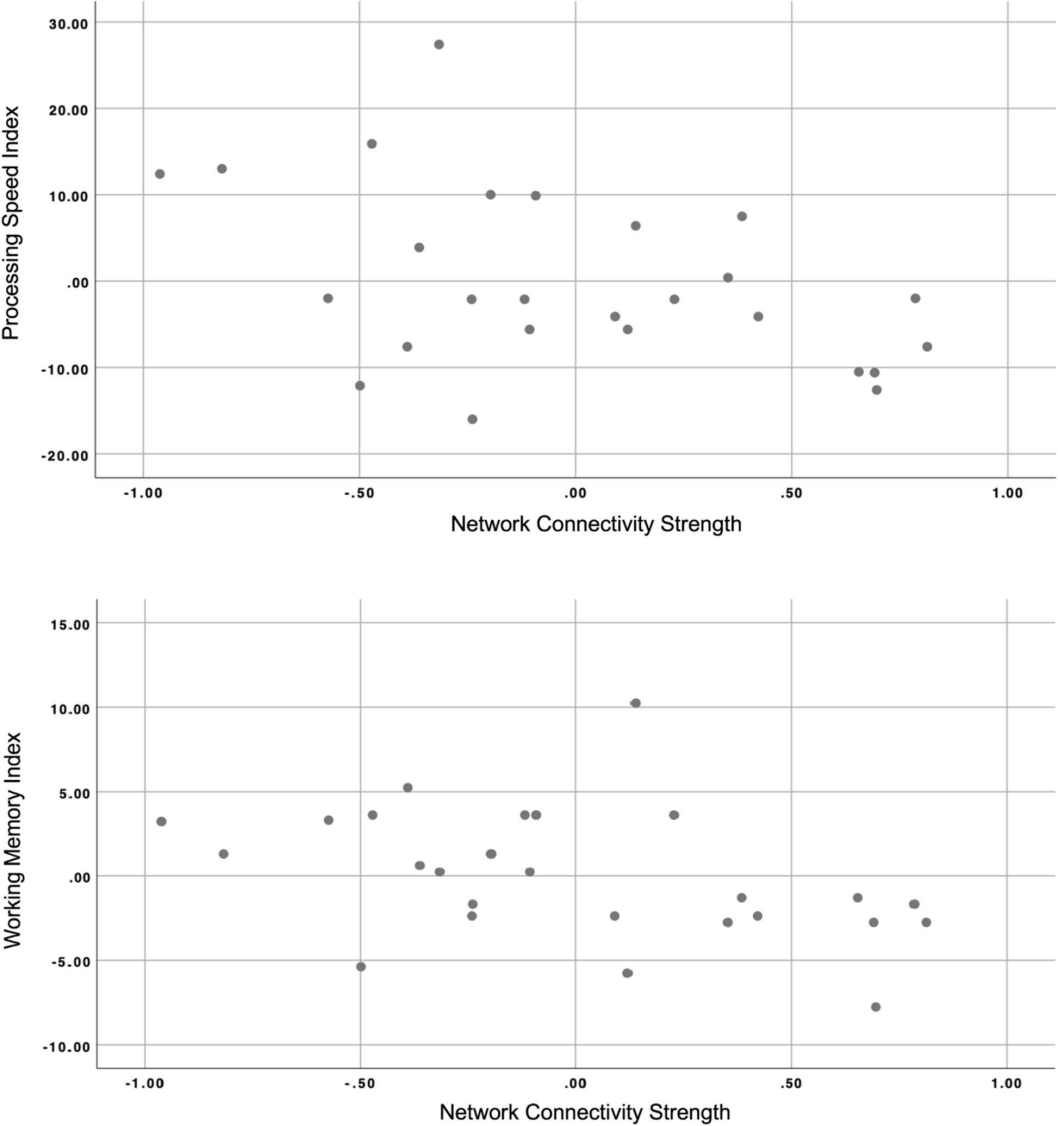
Scatter plots of partial regression results showing the association between network connectivity strength and processing speed (upper) and between network connectivity strength and the WM index (lower)

The linear regression analysis revealed a significant effect of interaction between network connectivity strength (β = -0.324; *p* = 0.041) and group (β = 1.124; *p* = 0.001) on the WM index (*R*^2^ = 0.822; *F* (3,22) = 33.901; *p* = 0.001). There was also a significant effect of interaction between the network connectivity strength (β = -0.687; *p* = 0.027) and group (β = 0.712; *p* = 0.024) on the processing speed index (*R*^2^ = 0.329; *F* (3/22) = 3.589; *p* = 0.030). Plots showing associations between network connectivity strength and WM and between network connectivity strength and processing speed are shown in Fig. 4.

## Discussion

This research aimed to explore whether the functional connectivity among the DMN, SN, FPN, and RNW structures differed between two groups of children with RLD: the LWM group and the AWM group. The results showed that individuals in the LWM group had significantly weaker connectivity in a network that comprises the rAG, lAG, rSMG, rIPL, and ACC than individuals in the AWM group. Importantly, the areas included in this network belonged to the DMN, SN, and FPN. Research has shown a functional interaction among these networks. For example, the SN regulates the connectivity between the DMN and the FPN, reflecting constant modulation between the resting and cognitive-processing states [21]. This network interaction has been related to cognitive functions, including WM and processing speed. The results are congruent with the literature, with higher WM scores related to stronger connectivity between the DMN and SN [35].

Furthermore, evidence indicates that TD children have higher functional connectivity than children with developmental dyslexia [22]. In the present study, it was expected that children with higher WM levels would have stronger connectivity. The obtained results further demonstrate the relation between the resting-state functional networks (DMN, SN, and FPN) and executive functions. Evidence shows that children with RLD had stronger connectivity among the superior frontal gyrus, middle frontal gyrus, and superior temporal gyrus than TD children during a reading comprehension task; this stronger connectivity seems to be related to poorer task performance and may compensate for the noted disability [49]. There is also resting-state fMRI evidence in an adult population of a positive relationship between DMN connectivity and reading comprehension, particularly the connectivity between the posterior cingulate cortex and anterior insula [50] and between the ACC and the middle temporal gyrus [51]. Although NBS analysis revealed a stronger connectivity of the resulting network in the AWM group, the regression analysis showed negative associations of the WM and processing speed indices with network connectivity strength. These results are congruent with the findings of Horowitz-Kraus et al. [49] that suggested that children with RLD have a compensatory mechanism independent of WM. Although a significant difference between groups in the specified RNW was not found in the present paper, the brain regions of the resulting network (supramarginal gyrus, angular gyrus, and inferior parietal lobe) are also related to the phonological process of reading [52] and could explain the findings of the linear regression analysis.

The domain-general hypothesis suggests that a deficit in WM would affect other higher-order cognitive processes, such as reading. Although we did not find significant differences in participants’ reading performance or in the functional connectivity within the specified reading-related network, other studies have included the angular gyrus, supramarginal gyrus and inferior parietal lobe as structures involved in the phonological process of reading [52]; these regions are relevant to the resulting network. Based on this, our results could link the cognitive processes of WM and phonological awareness. The lack of significant differences in reading abilities between the groups could be due to specific clinical difficulties in reading comprehension, reading accuracy, and reading speed experienced by individuals in both groups. Although both groups differed in their WM index, neither had an IQ below 75 on the WISC-IV.

Even though both groups had the same clinical diagnosis and reading impairment, analysis of resting-state fMRI data revealed a significant difference in how WM affected the functional connectivity of a cluster that included the DMN, SN, and FPN. Although the resulting network involved brain regions related to the phonological process of reading, the lack of significant differences between groups in the specified RNW yields inconclusive evidence regarding the domain-general hypothesis, under which a deficit in WM and other cognitive functions is proposed to impact complex cognitive functions, such as reading [8, 10]. According to Baddeley [17], WM has two slave systems: a phonological loop and a visuospatial sketchpad. The results prompt the question of which systems in WM are affected in children with RLD and whether they are related to other brain structures. Group comparison revealed connectivity strength differences in the angular gyrus, supramarginal gyrus, and inferior parietal lobe; such structures are also related to the phonological system in reading [52] and could play a role in the differences found in a network related to WM.

While the number of participants in this study is limited, the results pave the way for new considerations in children with RLD. On the one hand, we question whether WM differences affect more general factors embedded within fluid intelligence. Fluid intelligence comprises the cognitive processes related to inductive reasoning, reductive reasoning, and quantitative reasoning [53]. There is evidence supporting a relationship between executive functions and fluid intelligence [54]. A study conducted by Passolunghi et al. [55] with 182 fourth graders reported that the ability to solve inconsistent arithmetic word problems depended on the executive functions of WM, updating, and inhibition; this ability is not limited to solving arithmetic problems but also encompasses solving other problems, including verbal ones, that require fluid intelligence. WM and fluid intelligence differences may have a significant impact on reading comprehension even when accounting for children’s prior experience [56]. Although our AWM and LWM groups did not differ significantly in reading comprehension, this may be based on the specific difficulties of their RLD diagnosis that alter their overall reading performance. We also question whether these differences are related to other functions that may be affected, such as attention [12], or phonological consciousness, a key characteristic in reading [2]. On the other hand, the results raise questions regarding the clinical implications for children’s evaluation and treatment. Some training programs for children with RLD focus on domain-specific or domain-general functions. Aylward et al. [57] evaluated the behavioral and functional effects of instructional reading intervention in children with dyslexia. After three weeks of training, the authors observed that children had better phonological discrimination and reading comprehension that were related to higher activity in several brain structures, such as the cerebellum, inferior temporal gyrus, and inferior frontal gyrus. Notably, a study conducted by Ramezani et al. [58] focused on a visual WM intervention by considering affected variables, such as balance, resulting in a program that improved WM, reading skills, and postural control. There is also evidence that visual and verbal WM training applied separately or jointly improves performance on visual rhyming tasks, orthographic awareness, and fast word naming in children with DD [59].

Although many intervention programs focus on domain-specific or domain-general abilities, there are no clear criteria for selecting a specific training program. The results suggest that the WM index could be an additional variable for consideration in interventions because it is a factor in two subgroups of individuals with different functional relations between reading and WM-related brain structures; therefore, a distinct intervention program may be needed to address this factor.

## Conclusions

The results showed that cognitive differences in WM are associated with functional connectivity differences in a cluster that involves different functional brain networks (DMN, SN, and FPN). Even though the resulting network included brain regions related to the phonological process in reading activities, the lack of group differences in the connectivity of the specified RNW limits the support for the domain-general hypothesis. The results present new considerations for clinical evaluation and intervention. The consideration of other criteria, such as WM, could help or influence treatment decisions.

## Data Availability

The dataset supporting the conclusions of this article is available in the figshare repository, 10.6084/m9.figshare.19189889.
